# Effects of Myrtus (*Myrtus communis* L.) Extract Supplementation in the Diet on Metabolic, Immune, and Performance Parameters of Dairy Cows During the Transition Period

**DOI:** 10.3390/ani16040632

**Published:** 2026-02-16

**Authors:** Umit Ozcinar, Cangir Uyarlar, Muhammet Emre Orman, İbrahim Sadi Çetingül, Sababa Fatima, İsmail Bayram

**Affiliations:** Department of Animal Nutrition and Nutritional Diseases, Faculty of Veterinary Medicine, University of Afyon Kocatepe, ANS Campus, 03200 Afyonkarahisar, Türkiye; umitozcinar@gmail.com (U.O.); cangiruyarlar@hotmail.com (C.U.); sadicet@yahoo.com (İ.S.Ç.); sababafatima123@gmail.com (S.F.); ibayram1965@gmail.com (İ.B.)

**Keywords:** myrtus, transition, cows, milk, performance, blood profile, reproduction

## Abstract

This study evaluated the effects of dietary *Myrtus communis* L. extract on the production performance, immune functions, and metabolic and health status of Holstein cows throughout the transition period. Forty cows were assigned to either a control group (n = 20) or a treatment group (n = 20) receiving *Myrtus communis* L. extract at 1.5 mL/kg body weight per day. Milk yield and feed intake were recorded daily, whereas blood samples were collected weekly. Reproductive performance and health status were monitored. *Myrtus communis* L. supplementation increased dry matter intake during prepartum and early and mid-lactation and elevated prepartum glucose levels. Treated cows produced more milk in early (44.56 vs. 40.72 kg/day) (*p* < 0.0001), and mid-to-late lactation (42.57 vs. 37.19 kg/day) (*p* < 0.0001). NEFA and BHBA concentrations were lower in the treatment group (*p* = 0.015 and 0.019, respectively), whereas cortisol and ALT were higher around calving (*p* < 0.001). Despite similar prepartum inflammatory profiles, treated cows demonstrated higher TNF-α concentrations at calving (*p* = 0.006). The days open and insemination number per pregnancy did not differ; however, the number of ovarian cysts were less in treated cows. Overall, *Myrtus communis* L. extract offered at a dose of 1.5 mL/kg body weight during the transition period improved feed intake, enhanced milk production, and reduced metabolic stress, suggesting its potential as a natural additive to support energy balance and reproductive performance in cows. However, it increased the blood cortisol and ALT concentrations, which requires further investigation.

## Simple Summary

Dairy cows experience physiological and metabolic challenges during the transition period which can reduce feed intake, milk production, reproductive performance and metabolic homeostasis. The dietary interventions supporting metabolism and feed intake could have beneficial effects on improving milk yield, reproduction and the immune system. This study investigated the effects of dietary *Myrtus communis* extract supplementation on the performance, immune and metabolic parameters of Holstein cows during the transition period. *Myrtus communis* L. extract supplementation increased the feed intake and milk yield and improved the energy balance. Although some immune responses were more active around calving in supplemented cows, the overall health status was not negatively affected. Reproductive efficiency was similar between groups; however, cows supplemented with *Myrtus communis* L. extract developed fewer ovarian cysts. These findings suggest that *Myrtus communis* L. extract may help dairy cows by reducing the stress of calving, improving milk production, and supporting reproductive health.

## 1. Introduction

The transition period in dairy cattle, spanning three weeks before and after calving, represents one of the most physiologically demanding stages in the lactation cycle. During this period, cows experience dramatic shifts in metabolism, endocrine activity, and immunity, alongside increased nutrient requirements to support fetal growth and the onset of lactation [1]. These rapid physiological changes increase animals’ susceptibility to metabolic and infectious diseases, highlighting the need for nutritional and management strategies that support health and productivity [1,2].

Natural plant-derived compounds have emerged as promising nutritional interventions capable of enhancing gut health, immune competence, and oxidative stability [3,4]. Among these, herbal extracts are increasingly recognized for their capacity to improve digestive efficiency, support immune function, and reduce oxidative damage [5]. Phytogenic feed additives, defined as plant-derived bioactive substances incorporated into animal diets to enhance feed quality and animal performance, have been extensively investigated for their antimicrobial and antioxidant properties [6,7]. Supplementation with such phytogenic compounds has been shown to boost immune performance, modulate inflammation, and enhance antioxidant capacity in various animal species [7]. Their beneficial effects are attributed to multiple mechanisms, including enhanced nutrient absorption, the stimulation of digestive enzymes, the modulation of gut microbiota, and the improvement of systemic antioxidant defense [5].

*Myrtus communis* L. (myrtle) historically has been utilized in traditional medicine to treat a broad range of ailments, including respiratory infections, skin conditions, wounds, hemorrhoids, gastrointestinal disorders, and inflammatory diseases [8]. Contemporary pharmacological studies have confirmed the multifaceted therapeutic properties of *M. communis*, which include antioxidant, antimicrobial, antifungal, antidiabetic, hepatoprotective, anticancer, antiviral, and neuroprotective effects [5]. Animal studies have further demonstrated the potential benefits of *M. communis* essential oils and extracts. Improved performance outcomes have been documented in broilers [9], laying hens [10], rabbits [11], rats [12], calves [13] and quails [14]. The plant’s pharmacological effects are largely attributed to its abundance of bioactive constituents, including tannins, alkaloids, flavonoids, organic acids, and essential oils [5].

Despite the well-established pharmacological potential of *M. communis*, research examining its effects in ruminant nutrition remains limited. To our knowledge, few studies have evaluated its influence on the metabolic profile, immune status, and productivity of dairy cows. Given the metabolic stress and immune challenges faced by cows during the transition period, a dietary inclusion of *M. communis* extract may provide physiological support by reducing oxidative stress, stabilizing metabolism, and enhancing immune function. Therefore, the present study aimed to assess the effects of supplementing dairy cow diets with Myrtus communis extract during the transition period on production performance, metabolic indicators, and immune function.

## 2. Materials and Methods

### 2.1. Study Location and Animals

The research was conducted at a private Breeding Dairy Farm in Kapaklı Village, Burdur Province, Türkiye, at 30.2823° East Longitude and 37.7183° North Latitude. The study involved forty Holstein dairy cows weighing 615.7 kg in body weight and identified during their transition period, which encompassed the three weeks before the expected calving date and three weeks after calving. All animals were confirmed to be clinically healthy at the beginning of the study. The *Myrtus communis* L. supplementation was conducted throughout the transition period. However, feed consumption was measured until the 26th week after the myrtle extract supplementation was discontinued, while milk yield and some reproductive parameters were determined by collecting data from the herd monitoring program until the 42nd week. In addition, all cows were merged in a paddock and consumed the same diet for the remaining lactation days. Therefore, Myrtus extract supplementation in transition diets was the only variable for all cows throughout the lactation.

### 2.2. Experimental Design and Diet

The cows were randomly allocated into two equal groups: a control group (n = 20) and a treatment group (n = 20). The cows were fed in a single pen. Body condition score detection was not performed. But all cows were weighed for proper dosage decision, and body weight was similar for both groups. Lactation numbers and the number of multiparous/primiparous were similar for both groups. Each group had four primiparous and sixteen multiparous cows. Moreover, the groups had similar milk yields for the previous lactation. Animals in the control group were fed the basal total mixed ration (TMR) without any additional supplementation, whereas cows in the treatment group received *Myrtus communis* L. extract. Cows were housed in two separate pens, with each experimental group consisting of a single pen, and were subjected to group feeding. Total mixed ration (TMR) was offered simultaneously to both groups. Feed intake was recorded daily on a group basis by weighing the offered and refused feed. Average daily dry matter intake per cow was calculated by dividing group feed intake by the number of cows in each group. Subsequently, monitoring of feed intake was maintained from the end of week 4 up to week 26, when the supplementation period concluded.

The extraction process and chemical composition of the *Myrtus communis* L. extract was adopted from the Uyarlar et al. [13] and presented in Table 1. Myrtle extract was produced by a commercial company (Biyoderm^®^, ArsArthro Biotechnologies Inc., Ankara, Turkey). For extraction, dried myrtle plant (*Myrtus communis* L.) leaf and root were initially shredded using a mechanical shredder. The shredded parts were then immersed in sterile distilled water (1 part myrtle and 99 parts water) with a pH of 4.5 at 98 °C for 22 min. Afterward, the solution was allowed to cool, and its acidity with pH 4.5 was balanced using Na_2_HPO_4_ as a buffer solution, if needed. The resulting solution was filtered with medium flow, ash content < 0.01% and a thickness of 0.2 mm. Subsequently, it was bottled and stored in the refrigerator until needed.

A total of 18,471 L of myrtle extract (1.5 mL × 615.7 kg × 20 heads) was top dressed daily onto the TMR for each group. This application was done immediately after the feed was served to the animals. All cows were weighed at the beginning of the study. The dose of Myrtus extract was calculated based on the average live weight at the beginning of the study (615.7 kg). No further weighing was performed. The dosage protocol followed the recommendations outlined by Uyarlar et al. [13]. Both groups received an identical TMR formulated according to the nutrient requirements for transition dairy cows specified by the National Research Council [15]. Feed was offered once daily, and any refusals were collected and weighed prior to the next feeding to calculate the dry matter intake. Feed intake was recorded starting from the fourth week before Myrtus supplementation and continued through the fourth week postpartum. Subsequently, monitoring of feed intake was maintained from the end of week 4 up to week 26, when the supplementation period concluded. Nutrient composition of feed ingredients was analyzed according to the standard procedures of AOAC [16]. The chemical composition of the diets was determined at the beginning of the prepartum and postpartum periods. The ingredients and chemical composition of the prepartum and postpartum basal diets are presented in Table 2.

### 2.3. Health Monitoring and Reproductive Management

Following parturition, all cows were observed for the occurrence of common peripartum disorders, including hypocalcemia, retained fetal membranes, metritis, mastitis, and ketosis. For each animal, data recorded in the herd management system were used. All production and health-related parameters were individually recorded on electronic cow records within the herd management system, and the data were retrieved directly from this system. Any diagnosed condition was treated promptly according to standard veterinary protocols. Reproductive performance was evaluated through regular examinations conducted every 23 days to monitor uterine involution and ovarian follicular dynamics. Cows exhibiting behavioral or physical signs of estrus were artificially inseminated approximately 6–8 h after detection. Pregnancy diagnosis was performed using transrectal ultrasonography between 30 and 40 days post-insemination and reconfirmed two months later to identify possible embryonic losses. Fertility data, including pregnancy rates and embryonic mortality, were systematically recorded throughout the study period.

### 2.4. Milk Production and Blood Sampling

Milk yield for each cow was recorded daily throughout the experimental period using the farm’s automated herd monitoring system. Group milk production data were obtained from the same system and evaluated not only during the first four weeks postpartum but also continuously up to week 42 of lactation. Milk efficiency was calculated separately, dividing the milk yield by the body weight for 1–4 weeks and 4–42 weeks of lactation.

In addition, 10 mL blood samples were collected weekly from the coccygeal vein (vena coccygea) using vacuum tubes during the first eight weeks of the trial (−3, −2, −1, 0 [calving], +1, +2, +3, and +4 weeks). Immediately after collection, samples were centrifuged at 4200 rpm for 10 min to obtain serum, which was stored at −20 °C until subsequent biochemical analyses. NEFA, BHBA, glucose, total protein, total cholesterol, triglycerides, alanine aminotransferase, aspartate aminotransferase, gamma-glutamyl transferase, and blood urea nitrogen levels were determined using a spectrophotometer. On the other hand, cortisol (EA0021Bo), immunoglobulin G (IgG; E0010Bo), tumor necrosis factor-alpha (TNF-α; EA0009Bo), interleukin-6 (IL-6; E0001Bo), serum amyloid A (SAA; EA0012Bo), and haptoglobin (EA0011Bo) were determined using commercially available diagnostic kits (BT Lab, Shanghai, China) designed for use with a fully automated ELISA analyzer (Mindray Medical International Ltd., Shenzhen, China).

### 2.5. Statistical Analysis

Statistical analyses were performed using the JMP software (JMP, 2003, Version 5). The pen was considered as the experimental unit for dry matter intake on a weekly basis, allowing the comparison of seven data, whereas the individual cow was considered as the experimental unit for milk yield, blood, and immune parameters. Differences between the control and treatment groups were analyzed using the least square means method (LSM), with treatment (control vs. experimental) and sampling time included as fixed effects. The incidence of clinical health disorders was compared between groups using Pearson’s chi-square test. Variables potentially occurring multiple times within the same animal, such as the number of ovarian cysts, days open, and insemination frequency, were analyzed using independent sample *t*-tests. The probability value of *p* < 0.05 was considered indicative of statistical significance.

## 3. Results

### 3.1. Production Parameters

**Figure 1:**
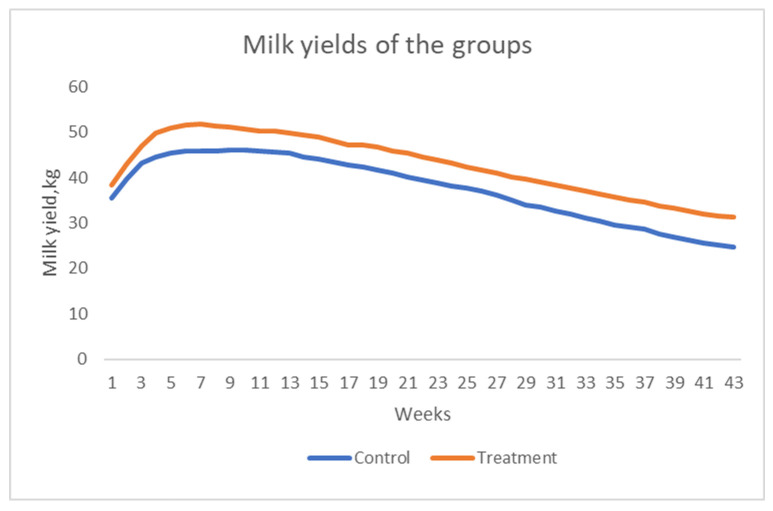
Effect of Myrtus on milk production.

The effects of Myrtus extract supplementation on milk yield are summarized in Table 3. During early lactation (1–4 weeks postpartum), cows receiving the extract produced significantly more milk than the control group (44.56 vs. 40.72 kg/day; *p* < 0.0001). This elevated production persisted through mid-to-late lactation (*p* < 0.0001). Also, supplementation improved the milk efficiency in early and early-to-mid-lactation. A significant effect of time was observed in both lactation periods (*p* < 0.0001), reflecting normal lactation trends. The interaction between treatment and time was not significant for either early (*p* = 0.8287) or mid-to-late lactation (*p* = 1.0000) (Figure 1).

**Figure 2:**
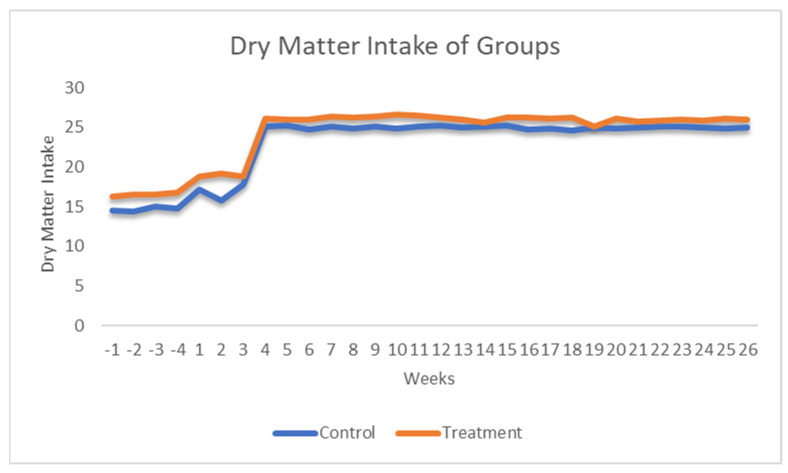
Effect of myrtus on dry matter intake.

Dry matter intake (DMI) differed significantly between groups across all evaluated periods (Table 3). During the prepartum phase, cows receiving Myrtus extract consumed more feed than control cows (*p* < 0.0001) (Figure 2). Neither time nor the treatment × time interaction had a significant effect during this phase (*p* > 0.05). In early lactation (parturition to +4 weeks), DMI increased in both groups, with supplemented cows again exhibiting a higher intake. Both treatment and time had significant effects (*p* < 0.0001), while the interaction was not significant (*p* = 0.1678).

During mid-lactation (+4 to +26 weeks), cows supplemented with Myrtus extract maintained a higher DMI relative to controls (*p* < 0.0001). In this period, the treatment effect remained significant, whereas the effects of time and the treatment × time interaction were not statistically significant (*p* > 0.05).

### 3.2. Reproduction Parameters

Reproductive parameters are summarized in Table 3. There was no significant difference between groups in the number of open days, with control cows averaging 185 days and cows supplemented with Myrtus extract averaging 180 days (*p* = 0.781). The average number of artificial inseminations per conception was slightly lower in the supplemented group compared to the control group; however, this difference did not reach statistical significance (*p* = 0.088). The number of confirmed pregnancies was higher in treated cows than in the control group, although the difference was not statistically significant.

### 3.3. Blood Parameters During Prepartum Period

Serum biochemical analysis indicated that cows supplemented with *Myrtus communis* L. extract exhibited lower non-esterified fatty acid (NEFA) concentrations compared to the control group (Table 4). While the treatment effect was not statistically significant (*p* = 0.130), time had a significant influence (*p* < 0.001). Similarly, beta-hydroxybutyrate (BHBA) levels were lower in the *Myrtus communis* L.-supplemented group (0.489 mmol/L) relative to the controls (0.525 mmol/L), with a significant effect of time (*p* < 0.004) and no significant treatment effect (*p* = 0.330). Glucose concentrations were elevated in the treated cows compared to the controls (*p* = 0.0.13).

No significant differences were observed between groups for total protein, BUN, triglycerides, total cholesterol, AST, and IgG. In contrast, GGT activity was significantly higher in supplemented cows than in controls (*p* = 0.005). Cortisol concentrations were also elevated in the *Myrtus communis* L. extract group. No significant differences were found between groups for TNF-α (*p* = 0.280), IL-6 (*p* = 0.630), serum amyloid A (*p* = 0.523), or haptoglobin (*p* = 0.323) (Table 4).

### 3.4. Blood Parameters at Parturition

At parturition, both NEFA and BHBA concentrations increased relative to prepartum values. Cows supplemented with *Myrtus communis* L. extract exhibited lower NEFA and BHBA concentrations compared to controls, with significant differences between groups. Glucose concentrations did not differ significantly between groups at parturition (*p* = 0.415) (Table 5).

ALT activity was significantly elevated in Myrtus-treated cows relative to the controls (*p* < 0.001). On the other hand, the cortisol level was higher in the treatment group compared to the control group. Other biochemical markers, including AST, GGT, and IgG, showed no significant differences between groups at calving. TNF-α levels were significantly higher in the supplemented group compared to the control group. In contrast, IL-6, serum amyloid A, and haptoglobin concentrations did not differ significantly between groups (*p* > 0.05) (Table 5).

### 3.5. Blood Parameters During Postpartum Period

In the postpartum period, both NEFA and BHBA concentrations decreased relative to prepartum levels. Nonetheless, these metabolites remained significantly higher in the control group compared to cows supplemented with *Myrtus communis* L. extract (Table 6). Blood glucose concentrations were slightly but significantly higher in the *Myrtus communis* L.-treated cows relative to controls. Postpartum levels of triglyceride, total protein, total cholesterol ALT, AST, GGT and cortisol were similar between groups (*p* > 0.05). There was a trend to increased levels of IgG in the supplemented groups (*p* = 0.052). Supplemented cows exhibited significantly higher concentrations of TNF-α (*p* = 0.0017), IL-6 (*p* = 0.0027), and serum amyloid A (*p* = 0.0022) compared to the controls. Haptoglobin levels, however, were not significantly affected by the treatment (*p* = 0.2217).

### 3.6. Incidence of Health Disorders

The incidence of reproductive and health disorders in control and *Myrtus communis* L.-supplemented cows is summarized in Table 7. Although the number of animals that experienced postpartum diseases such as placental retention, mastitis, metritis, displaced abomasum, acidosis, and ketosis was lower in the Myrtus group, no statistically significant differences were observed between the groups (*p* > 0.05). The incidence of ovarium cysts was lower in the supplemented group (*p* = 0.031). Also, there was a trend for reduced embryonic death in supplemented cows (1 case vs. 6 cases; *p* = 0.091).

## 4. Discussion

### 4.1. Performance Parameters

The presented study indicates that *Myrtus communis* L. supplementation exerted multiple beneficial effects, particularly regarding milk yield, dry matter intake (DMI) and energy metabolism.

*Myrtus communis* L. supplementation significantly increased milk production during the early and mid-lactation period. These improvements in dry matter intake and milk yield are compatible with findings from other phytogenic feed additives, whose beneficial effects have been attributed to their bioactive content such as polyphenolic polymers leading to enhanced rumen fermentation, antioxidant activity, nutrient use efficiency, and improved lactational performance [17,18]. Dry matter intake is a primary driver of milk production, and increases in DMI are well known to support a higher milk yield. Previous studies demonstrated that an improved nutrient intake leads directly to a greater milk output, as enhanced DMI provides the additional energy and substrates required for lactation [19]. The exact mechanism of the *Myrtus communis* L. supplementation on the increase in dry matter intake is not clearly explained. However, it is considered that the *Myrtus communis* L. extract may exert its effects through essential oils, flavonoids, and tannins, which modulate ruminal microbial populations, improve fiber degradation, suppress undesirable microbial populations, and enhance feed conversion efficiency [20,21]. These effects likely contributed to the observed increases in both milk production and DMI. There are different findings regarding the effects of *Myrtus communis* L. on feed consumption in other animal species [22,23]. Salehifar et al. [22] reported that *Myrtus communis* L. oil increased feed consumption in broiler chickens, and that this effect was due to *Myrtus communis* L. stimulating endogenous digestive enzymes, thereby increasing total nutrient digestion and digestion rate.

Additionally, Saei et al. [23] reported that, when *Myrtus communis* L. oil was added to the ration of broilers severely exposed to aflatoxicosis, some blood biochemical parameters (serum glucose, creatinine, cholesterol, ALT, AST, ALP) improved, and feed consumption and live weight gain increased. Accordingly, it is thought that *Myrtus communis* L. may be beneficial in conditions when the risk of health disorders is high, especially those that strain liver metabolism. Therefore, the use of *Myrtus communis* L. extract in this study during the transition period, when energy metabolism and liver performance are most affected in dairy cows, may be useful in explaining this increase in feed consumption and the resulting increase in milk yield.

### 4.2. Reproductive Health

In the present study, *Myrtus communis* L. extract supplementation led to numerical improvements in reproductive performance parameters such as a higher pregnancy rate and a reduced number of artificial inseminations per pregnancy. Similarly, the days open did not differ significantly between the control and treatment groups, suggesting that *Myrtus communis* L. extract may not have a substantial impact on estrus duration or calving interval under the conditions of this experiment. Nevertheless, evidence from comparable studies suggests that *Myrtus communis* L. extract and other herbal feed additives can positively influence reproductive physiology. Uyarlar et al. [13] reported that supplementation with *Myrtus communis* L. extract reduced the interval between birth to first insemination and decreased the number of inseminations required for conception in Holstein calves. Phytogenic constituents from medicinal plants are known to exhibit hormone-like activity, mimicking natural estrogens and improving fertility in both male and female livestock animals. For example, Emre et al. [24] demonstrated that *Momordica charantia* L. extract enhanced reproductive performance by elevating insulin-like growth factor-1 (IGF-1) levels in repeat breeder cows, particularly those with subclinical endometritis (SCE), thereby improving conception rates. Similarly, Barlas et al. [25] observed that oral myricetin administration (100 mg/day) induced estrogenic responses in immature Wistar albino rats, reflected by an increased uterine weight and length compared to control animals receiving ethinyl estradiol, ethinyl estradiol plus tamoxifen, or genistein. Additional studies by Abidli et al. [26] indicated that phenolic compounds in *Myrtus communis* L. enhance reproductive function in both male and female animals, while Vakili et al. [27] reported that dietary myrtle leaf powder (25 g/kg) improved reproductive performance, seminal plasma antioxidant capacity, and blood superoxide dismutase (SOD) activity in Arabian rams. These effects were attributed to enhanced uterine tone, improved uterine blood flow and microcirculation, and strengthened uterine immunity [28]. Collectively, these findings indicate that *Myrtus communis* L. extract and other phytogenic compounds may support reproductive performance through mechanisms including hormonal modulation, antioxidant activity, and improved uterine health. While the present study did not detect statistically significant improvements in reproductive indices, the observed trends, together with the supporting literature, suggest that *Myrtus communis* L. extract has potential as a natural enhancer of reproductive efficiency in dairy cows.

### 4.3. Blood Metabolites During Transition Period

#### 4.3.1. Prepartum Phase

During the prepartum period, a supplementation with *Myrtus communis* L. extract positively influenced several metabolic and physiological parameters, suggesting an improved adaptation to the metabolic alterations in the transition period. In cows treated with Myrtus communis L. extract, concentrations of unesterified fatty acids and β-hydroxybutyrate were numerically lower, while glucose concentrations were slightly higher; this indicates that the overall carbohydrate metabolism remained stable across all treatments.

Systemic inflammation parameters such as serum TNF-α, IL-6, serum amyloid A, and haptoglobin were similar between groups. These findings are consistent with the studies on immune homeostasis of dairy cows in the prepartum period [20,29]. Bioactive molecules of *Myrtus communis* L. extract, such as flavonoids and phenolic compounds, have been shown to possess anti-inflammatory and antioxidant properties [20]. Previous studies have demonstrated that phytogenic feed additives can reduce inflammatory responses in ruminants by downregulating cytokine expression or lowering oxidative stress [30]. Thus, the current findings indicate that, while *Myrtus communis* L. extract supplementation had no significant effect on inflammatory or acute-phase markers, it has the potential to maintain physiological immunological balance and minimize subclinical inflammation.

The combination of improved energy metabolites (lower NEFA and BHBA, slightly higher glucose) and normal cytokine levels indicates that Myrtus may support metabolic stability and immune readiness prior to parturition.

#### 4.3.2. Parturition Phase

Serum NEFA and BHBA concentrations were elevated in both control and *Myrtus communis* L. extract-supplemented cows at the parturition. These findings are consistent with the theory of body fat mobilization to meet the increased energy demands associated with calving and the onset of lactation. These changes are consistent with the well-documented signs of negative energy balance that occur in transition cows, driven by the sudden increase in nutrient requirements for milk synthesis and the limited feed intake around calving [31].

*Myrtus communis* L. supplementation significantly reduced NEFA concentration. The lower NEFA and BHBA levels in the treatment group indicate the improvement of energy metabolism, indicating a reduced adipose tissue mobilization and ketone body production in the liver, suggesting a decreased risk of metabolic disorders such as ketosis and fatty liver syndrome during early lactation. These findings are supported by previous research demonstrating that phytogenic feed additives can improve energy balance by enhancing rumen fermentation efficiency and modulating lipid mobilization [17,18]. Glucose concentrations did not differ significantly between groups. This suggests that *Myrtus communis* L. extract does not interfere with carbohydrate metabolism at calving, despite its effects on lipid mobilization. There were increased levels of cortisol and ALT and cortisol levels in treated cows around parturition. It is difficult to explain these increases, since there were no any adverse effects on the other blood metabolites and disease incidence, increasing the need for further studies.

*Myrtus communis* L. extract supplementation led to a considerable increase in blood TNF-α concentration, whereas IL-6, serum amyloid A, and haptoglobin levels remained stable. Phytogenic feed additives can alter cytokine release through antioxidant or immunomodulatory activities, depending on the dose, duration, and physiological status [30]. The slight increase in TNF-α could therefore reflect immunological activation or enhanced alertness [32], while acute-phase proteins (serum amyloid A and haptoglobin) remained unaltered. It is possible that the animals in the current study were not subjected to significant inflammatory or metabolic stress, limiting the observable anti-inflammatory effect of *Myrtus communis* L. extract. These findings suggest that *Myrtus communis* L. extract may exert a minor immunomodulatory effect, characterized by a slight increase in proinflammatory cytokine activity without causing systemic inflammation.

#### 4.3.3. Postpartum Phase

Following calving, both control and *Myrtus communis* L. extract-supplemented cows exhibited a decline in NEFA and BHBA concentrations compared with periparturient values, reflecting the normal recovery of energy balance in early lactation. However, NEFA and BHBA levels remained significantly lower in the *Myrtus communis* L. group. These findings suggest that supplementation facilitated better metabolic adaptation during the transition phase and may reduce the risk of ketosis or fatty liver.

These results are consistent with previous studies showing that phytogenic feed additives, including herbal extracts, can improve hepatic function, modulate lipid metabolism, and support antioxidant defense in transition cows [17]. Overall, *Myrtus communis* L. extract supplementation during the transition phase appears to enhance energy balance, support hepatic efficiency, and stabilize key metabolic parameters without inducing adverse effects. Cows treated with *Myrtus communis* L. extract had significantly increased postpartum concentrations of TNF-α, IL-6, and serum amyloid A compared to the control group, whereas haptoglobin levels were similar. This increase in proinflammatory cytokines and acute-phase proteins could be attributed to a minor immune system activation in response to parturition and the initiation of lactation, which are often linked with transitory inflammatory stress [33].

Myrtus includes bioactive substances such as flavonoids, terpenoids, and phenolic acids, which might influence cytokine expression and antioxidant defense mechanisms [20]. Such substances may increase macrophage activity and cytokine signaling pathways, helping to boost immunological adaptation during early lactation [34]. Similar trends have been observed in studies in which phytogenic additives or essential oils increased cytokine secretion and acute-phase responses under metabolic or oxidative stress, allowing animals to better adapt to the physiological challenges of the transition period [30]. The elevated serum amyloid A concentration seen here supports this claim, as SAA is an early and sensitive indicator of immune activation and tissue healing [29]. Overall, our findings suggest that *Myrtus communis* L. extract supplementation may induce a balanced increase in postpartum immune activity, thereby aiding recovery, uterine health, and metabolic adaptation following calving.

### 4.4. Disease Incidence

There were no significant differences between groups for the incidence of retained placenta, metritis, mastitis, displaced abomasum, acidosis, or ketosis. However, the number of ovarian cysts was significantly lower in the treatment group. Furthermore, there was a trend toward fewer embryonic deaths among the treated cows. While differences in other health parameters were not statistically significant, these results suggest that *Myrtus communis* L. extract supplementation may help reduce the occurrence of ovarian cysts and potentially support improved reproductive outcomes. The antioxidant and endocrine-modulating capabilities of *Myrtus communis* L. extract bioactives, such as flavonoids, phenolic acids, and essential oils, may explain the considerable decrease in ovarian cyst incidence [20]. These substances have been shown to affect ovarian function by regulating oxidative stress, steroidogenesis, and luteal activity [35]. By enhancing the oxidative and metabolic environment, *Myrtus communis* L. extract supplementation may have maintained normal follicular growth and ovulatory cycles, minimizing the risk of cystic ovarian degeneration. The observed patterns of decreased incidences of metritis, mastitis, and embryonic loss are consistent with the anti-inflammatory and immune-stabilizing effects of phytogenic additions in ruminants [30]. Improved feed intake and metabolic adaptability, as seen in *Myrtus communis* L.-supplemented cows in the current study, may have indirectly contributed to better postpartum recovery and reproductive success. Overall, these findings indicate that *Myrtus communis* L. extract supplementation may reduce the incidence of postpartum reproductive and metabolic diseases, owing to its antioxidant, anti-inflammatory, and immunomodulatory activities.

The present study investigated the effect of *Myrtus communis* L. extract supplemented throughout the transition period on the transition period and its carryover effect on the early-to-mid-lactation performance and blood and immune markers. Considering the short duration of supplementation, further studies adopting a longer supplementation are needed to better understand the effect of *Myrtus communis* on the full lactation cycle. Additionally, in this study, cows were divided into two groups, and there was no individual feeding. Further studies determining the individual feed intake and supplement intake are needed to better understand its effect on feed intake and feed use efficiency.

## 5. Conclusions

This study found that supplementation with *Myrtus communis* L. extract with a dose of 1.5 mL/kg live weight per animal can improve several key parameters in dairy cattle during the transition period. In particular, *Myrtus communis* L. extract increases milk production and DMI and improves energy metabolism by lowering NEFA and BHBA levels. It did not affect the reproductive parameters, excluding reduced ovarium cysts in treated cows. Although there were positive trends in the pregnancy rate and embryonic viability in treated cows, these data did not show statistical significance. Further studies elucidating the hormonal, microbial and metabolic mechanisms are needed to better understand the effect of *Myrtus communis* L. on dairy cows.

## Figures and Tables

**Table 1:** Chemical composition of the *Myrtus communis* L. extract.

Amino Acid Profile		Mineral Profile	
Amino Acid	Amount (mg/L)	Mineral	Amount (mg/L)
Proline	16,254.60	Sodium	324,000
Glutamic acid	1767.92	Magnesium	27,000
Histidine	1133.84	Aluminum	1.35
Tyrosine	1003.04	Iron	7.10
Tryptophan	907.13	Tin	1.00
Alanine	691.27	Lead	0.007
Aspartic acid	668.01	Cadmium	0.002
Arginine	634.54	Mercury	0.03
Glycine	417.49	Arsenic	0.003
Isoleucine	414.90	**Phenolic Profile**	
Cystine	218.82	Phenolic Compound	Amount (mg/L)
Methionine	281.53	Myricetin	15.34
Serine	182.91	Catechin	4.80
Leucine	173.50	Quercetin	0.19
Valine	151.64	Gallic acid	0.13
		Salicylic acid	0.06
		Rosmarinic acid	0.01

**Table 2:** Basal diet (TMR) ingredients.

Ingredients			Chemical Composition		
	Prepartum, % DM Basis	Postpartum, % DM Basis		Prepartum	Postpartum
Corn silage	24.79	26.15	NEL (Mcal/kg)	1.38	1.71
Dried alfalfa	13.65	17.64	Crude Protein (DM%)	12.59	16.04
Wheat straw	24.34	0	Crude fat (DM%)	3.52	4.96
HMCG	11.82	13.14	Ash. (D%M)	8.46	9.06
DDGS	8.05	8.95	NDF (DM%)	41.75	28.94
Corn flake	7.73	17.39	ADF (DM%)	27.62	17.76
Soybean meal (47% CP)	5.86	11.64	Calcium (DM%)	0.98	0.83
CSFA	0	1.90	Phosphorus (DM%)	0.37	0.37
Salt	0.38	0.47			
Calcium Chloride	2.09	1.35			
Premix	1.29	1.37			

HMCG: High-moisture corn grain; DDGS: Distillers Dried Grains Solubles; CSFA: Calcium salt of fatty acids; Coated Calcium Chloride (NutriCab, Kemin); provided per kg of Premix: 350,000 IU of Vitamin A, 50,000 IU of Vitamin D, 900 mg of Vitamin E, 1200 mg of Mn, 500 mg of Fe, 500 mg of Zn, 900 mg of Cu, 40 mg of I, 10 mg of Co. Neutral detergent fiber; acid detergent fiber.

**Table 3:** Effect of Myrtus on performance and reproduction parameters.

	Groups			P		
	Control	Myrtus	SEM	Treatment	Time	Treatment × Time
Milk yield, kg (1–4 weeks)	40.72	44.56	0.6736	<0.0001	<0.0001	0.8287
Milk yield, kg (5–42 weeks)	37.19	42.57	0.1945	<0.0001	<0.0001	1.0000
DMI, kg/per animal (−4 weeks-parturition)	14.70	16.47	0.1796	<0.0001	0.7723	0.8789
DMI, kg/per animal (parturition +4 weeks)	19.30	20.73	0.1412	<0.0001	<0.0001	0.1678
Milk efficiency (1–4 weeks)	0.066	0.072				
Milk efficiency (5–42 weeks)	0.060	0.069				
DMI, kg/per animal (+4–+26 weeks)	24.99	26.12	0.0483	<0.0001	0.7044	0.4701
Open days	184.29	180.10	11.099	0.7829	-	-
Artificial insemination	2.95	2.55	0.1618	0.0886	-	-
Number of pregnant animals	16	18				

Data are represented as least square means. Control = no supplement. Myrtus = Mrytus extract top dress supplementation to TMR; standard error of means; dry matter intake.

**Table 4:** Effect of *Myrtus communis* L. on prepartum blood parameters (−4 week parturition).

	Groups			P		
	Control	Myrtus	SEM	Treatment	Time	Treatment × Time
NEFA, mmol/L	0.53	0.48	0.024	0.130	<0.001	0.288
BHBA, mmol/L	0.52	0.48	0.025	0.330	<0.004	0.099
Glucose, mg/dL	45.05	47.49	0.686	0.013	0.504	0.027
Total protein, g/dL	6.56	6.62	0.048	0.405	0.865	0.928
BUN, mg/dL	11.21	11.44	0.119	0.189	0.009	0.177
Triglyceride, mg/dL	16.58	16.88	0.464	0.644	0.047	0.986
Total cholesterol, mg/dL	105.39	122.74	2.738	0.060	0.053	0.104
ALT, U/L	18.74	18.95	0.145	0.307	0.260	0.065
AST, U/L	68.52	66.79	0.563	0.032	0.665	0.588
GGT, U/dL	18.29	19.45	0.287	0.005	0.007	0.003
IgG, mg/mL	22.02	21.35	0.356	0.357	0.190	0.443
Cortisol, µg/dL	23.31	25.27	0.648	0.034	0.010	0.327
TNFα, pg/mL	83.39	82.29	0.709	0.280	-	-
IL-6, pg/mL	108.71	109.28	0.841	0.630	-	-
Serum amyloid A, mg/L	14.30	13.85	0.501	0.523	-	-
Haptoglobin, mg/L	127.29	133.50	4.383	0.323	-	-

Data are represented as least square means. Control = no supplement. Myrtus = Mrytus extract top dress supplementation to TMR; standard error of means; non-esterified fatty acids; beta hydroxy butyric acid; blood urea nitrogen; alanine aminotransferase; aspartate aminotransferase; gamma glutamyl transferase; immune globulin G; tumor necrosis factor; interleukin-6.

**Table 5:** Effect of *Myrtus communis* L. on parturition blood parameters.

	Groups			
	Control	Myrtus	SEM	P
NEFA, mmol/L	1.16	0.84	0.088	0.015
BHBA, mmol/L	1.09	0.78	0.092	0.019
Glucose, mg/dL	41.40	43.33	1.663	0.415
Total protein, g/dL	6.80	6.72	0.080	0.494
BUN, mg/dL	11.46	11.59	0.236	0.688
Triglyceride, mg/dL	9.38	9.68	0.553	0.699
Total cholesterol, mg/dL	127.29	127.17	5.214	0.987
ALT, U/L	18.88	22.28	0.433	<0.001
AST, U/L	86.82	81.61	2.465	0.140
GGT, U/dL	29.49	28.61	1.311	0.638
IgG, mg/mL	19.30	18.36	0.466	0.166
Cortisol, µg/dL	28.79	37.80	1.313	<0.001
TNFα, pg/mL	86.89	90.56	0.919	0.006
IL-6, pg/mL	110.61	112.82	0.817	0.063
Serum amyloid A, mg/L	16.81	17.29	0.2511	0.1798
Haptoglobin, mg/L	149.94	149.48	4.4523	0.9428

Data are represented as least square means. Control = no supplement. Myrtus = Mrytus extract top dress supplementation to TMR; standard error of means; non-esterified fatty acids; beta hydroxy butyric acid; blood urea nitrogen; alanine aminotransferase; aspartate aminotransferase; gamma glutamyl transferase; immune globulin G; tumor necrosis factor; interleukin 6.

**Table 6:** Effect of *Myrtus communis* L. on postpartum blood parameters (parturition—4 weeks).

	Groups			P		
	Control	Myrtus	SEM	Treatment	Time	Treatment × Time
NEFA, mmol/L	1.18	0.91	0.054	0.0006	0.1804	0.793
BHBA, mmol/L	1.13	0.74	0.051	<0.001	0.9105	0.1093
Glucose, mg/dL	45.53	47.82	0.591	0.0071	0.020	0.3418
Total protein, g/dL	7.55	7.44	0.068	0.2357	0.7791	0.5173
BUN, mg/dL	17.23	17.16	0.373	0.9065	0.9673	0.5725
Triglyceride, mg/dL	9.67	9.98	0.316	0.485	0.0702	0.0064
Total cholesterol, mg/dL	160.07	163.73	3.739	0.490	0.532	0.578
ALT, U/L	21.62	21.80	0.318	0.688	0.5922	0.2935
AST, U/L	78.86	79.24	1.131	0.809	<0.001	0.2626
GGT, U/dL	26.80	26.39	0.678	0.669	0.0004	0.2390
IgG, mg/mL	19.34	20.56	0.438	0.0522	0.3083	0.4613
Cortisol, µg/dL	29.26	27.73	0.808	0.184	<0.001	0.6514
TNFα, pg/mL	90.06	94.66	0.9642	0.0017	-	-
IL-6, pg/mL	108.03	110.67	0.5796	0.0027	-	-
Serum amyloid A, mg/L	17.32	18.41	0.2357	0.0022	-	-
Haptoglobin, mg/L	152.72	160.31	4.3178	0.2217	-	-

Data are represented as least square means. Control = no supplement. Myrtus = Mrytus extract top dress supplementation to TMR; standard error of means; non-esterified fatty acids; beta hydroxy butyric acid; blood urea nitrogen; alanine aminotransferase; aspartate aminotransferase; gamma glutamyl transferase; immune globulin G; tumor necrosis factor; interleukin-6.

**Table 7:** Effect of *Myrtus communis* L. on incidence of health disorders.

Item	Control	Myrtus	P
Placental retention	4	2	0.661
Metritis	8	3	0.155
Mastitis	5	2	0.129
Displaced abomasum	0	0	-
Acidosis	3	2	1.00
Ketosis	2	0	0.487
Ovarium cyst	1	0.40	0.031
Embryonic death	6	1	0.091

## Data Availability

The raw data supporting the conclusions of this article will be made available by the authors on request.
